# An Efficient Synthesis of Arylated Pyridines from Conjugated Acetylenes and Substituted Benzylamines Catalyzed by Base

**DOI:** 10.3390/molecules22081277

**Published:** 2017-07-31

**Authors:** Mengping Guo, Bo Chen, Qiming Zhu, Hua Jin, Qiuling Peng, Yanping Kang

**Affiliations:** 1 College of Chemistry and Bio-Engineering, Yichun University, Yichun 336000, Jiangxi, China; chenbo25719@163.com (B.C.); Zhqm18@163.com (Q.Z.); 18079573672@163.com (H.J.); ycxypql@163.com (Q.P.); mkyp@sina.com (Y.K.); 2 Engineering Center of Jiangxi University for Lithium Energy, Yichun University, Yichun 336000, Jiangxi, China

**Keywords:** arylated pyridines, synthesis, transition-metal-free, base, catalysis

## Abstract

An efficient base-catalyzed synthesis of arylated pyridines has been disclosed. This reaction involving conjugated acetylenes and substituted benzylamines proceeded smoothly, giving rise to tri-aryl substituted pyridines which are biologically relevant compounds in good to excellent yields in *N*,*N*-dimethylformamide (DMF) under air at 140 °C with K_2_CO_3_ as catalyst.

## 1. Introduction

The importance of pyridine motif comes from its unique biological activity in natural products [1,2,3], pharmaceutical compounds [4,5,6,7,8] and agrochemicals [9]. In addition, pyridine derivatives are widely applied in organometallic chemistry [10,11], catalysis [12], material science [13,14,15] and supramolecular chemistry [16,17,18]. Therefore, the more efficient synthesis of pyridine derivatives is still an important topic [19,20]. However, there are only very few examples reported on this topic: in 1974, Chalk [21] reported a new pyridine synthesis from conjugated acetylenes and substituted methylamines, leading to 51% of 2-*p*-tolyl-3,6-diphenylpyridine and 38% of 2-*p*-tolyl-3,6-diphenylpyridine N-oxide at 145 °C under nitrogen with dimethylsulfoxide as solvent. In 2013, Shaand coworkers [22] disclosed a facile synthetic method for the preparation of trisubstituted pyridines with high regioselectivity through a three-component assembly strategy of arynes, isocyanides, and 3-bromo- or 3-acetoxypropynes, leading to 65% of 2-(4-fluorophenyl)-3,6-diphenylpyridine. In recent years, transition-metal-catalyzed C-C cross-coupling reaction has been applied to a diverse array of fields. Peter [3] recently reported the site-selective arylation of commercially available 2,3,5,6-tetrachloropyridine using the Suzuki–Miyaura reaction, allowing the selective synthesis of mono-, di-, tri- and tetraarylated pyridines in good to quantitative yields. In this context, based on the advantages of conjugated acetylenes, which are readily prepared by the catalytic oxidative coupling of terminal alkynes [23], studying more efficient synthesis of pyridine derivatives between conjugated acetylenes and substituted methylamines is still highly desirable and challenging.

## 2. Resultsand Discussion

Our interest in increasing the synthetic yield of arylated pyridines from conjugated acetylenes and substituted benzylamines under optimum conditions stemmed from the fact that Chalk’s [24] work gave only a 70% yield of 2,3,6-triphenylpyridine fromsolutions of 1,4-diphenylbutadiyne in benzylamine (1:6.13 mmol) after two to three hours at 180 °C under nitrogen. Initially, we tested the reaction of 1,4-diphenylbutadiyne **1** (1 mmol) and benzylamine **2** (6 mmol) in DMSO at 140 °C in the presence of K_2_CO_3_ (0.5 mmol) under air. To our delight, 2,3,6-triphenylpyridine **3c** was obtained in 85% isolated yield (Table 1, entry 3). Then, the effects of the ratio of starting materials **1**:**2** were examined (Table 1, entries 1–5). The yield of **3** improved to 96% with a **1**:**2** ratio of 1:8 or 1:10 (Table 1, entries 1–2). This result really encouraged us and extensive exploration of the conditions was further carried out. When the reaction temperature was dropped from 120 °C to 80 °C, 70% and 30% of the desired product **3** were obtained respectively (Table 1, entries 6–7). Subsequent solvent screening suggested that *N*,*N*-dimethylformamide (DMF) was the optimal one with 1,4-diphenylbutadiyne **1** (1 mmol) and benzylamine **2** (8 mmol) catalyzed by K_2_CO_3_ (0.5 mmol), and the desired product **3** was obtained in 99% isolated yield without any byproducts at 140 °C under air. It is worth noting that the reaction could proceed without a base, also as a catalyst, rendering the desired product in 38% isolated yield (Table 1, entry 11), which demonstrated that the yield of desired product **3** depends on the catalytic activity of the base. To demonstrate the catalytic value of a variety of bases, the synthetic reactions of 2,3,6-triphenylpyridine between 1,4-diphenylbutadiyne **1** (1 mmol) and benzylamine **2** (8 mmol) were carried out in DMF using different bases at 140 °C for 10 h with 0.5 mmol catalyst loading under air (Table 1, entries 12–20). The almost quantitative yield (99%) was obtained by using K_2_CO_3_ as the catalyst (Table 1, entry 8). Use of other bases, such as Na_2_CO_3_, NaOH, KOH and KHCO_3_ also gave good yields (Table 1, entries 13–15, 17). Under similar reaction conditions, Cs_2_CO_3_, NaF, NaH_2_PO_4_, KH_2_PO_4_ and CH_3_COONa afforded only moderate yield (Table 1, entries 12, 16, 18–20). These resultsindicate that K_2_CO_3_ is very effective in promoting the synthesis of arylated pyridines from conjugated acetylenes and substituted benzylamines under facile conditions.

Under the optimized reaction conditions, the scope of this synthetic protocol was evaluated to test the compatibility of varying symmetrical 1,4-diarylbuta-1,3-diynes as starting materials (Table 2). The 1,4-diarylbuta-1,3-diyne bearing two methyl groups at the 1- and 4-position was easily converted to give the desired products with excellent yield (90%) in the synthesis of arylated pyridines using benzylamine (**3cbb**). However, 1,4-bis(4-butylphenyl)buta-1,3-diyne was slightly less reactive, giving the desired product with 60% yield under the same conditions, and this result clearly demonstrated that steric hindrance has an effect on the yield of desired product (**3cfb**). The reaction using sterically hindered 1,4-di-*o*-tolylbuta-1,3-diyne and 1,4-di-*m*-tolylbuta-1,3-diyne led to 77% and 78% yields, respectively (**3ccb**, **3cdb**).Investigations of substituted benzylamines in the synthesis of arylated pyridines using 1,4-diphenylbutadiyne were also conducted. The reaction with substituted benzylamine having an electron-donating group was carried out efficiently, affording almost quantitative yield (99%) (**3cac**).Various substituted benzylamines bearing electron-withdrawing groups, such as -F, -Cl, and -CF_3_, provided the corresponding products in moderate to good yields (**3cad**, **3cae**, **3caf**). The steric and electronic effects of the substrate bearing electron-withdrawing substituent in the 3-position of benzylamine remarkably affected the reaction yield: upon using [3-(trifluoromethyl)phenyl]methanamine, product 3,6-diphenyl-2-[3-(trifluoromethyl)phenyl]pyridine was obtained in 50% yield (**3caf**).

## 3. Materials and Methods 

### 3.1. General Conditions

All manipulations were performed under air. All reagents employed in the synthesis were analytical grade, purchased from J&K Scientific Ltd. (Shanghai, China) and used as received without any prior purification. The products were isolated by thin layer chromatography on silica gel using petroleum ether as the eluent. ^1^H-NMR, ^13^C-NMR spectra were recorded on a Bruker Avance III (400 MHz, Bruker Corporation, Billerica, MA, USA) spectrometer using tetramethylsilane as the internal standard and CDCl_3_ as the solvent. Chemical shift values are expressed in ppm relative to external TMS (see Supplementary).

### 3.2. General Procedure for the Preparation of Arylated Pyridines

1,4-Disubstituted-1,3-diacetylene (0.25 mmol) and K_2_CO_3_ (0.5 mmol) were added, under air, to a solution of appropriate benzylamine (2.0 mmol) in DMSO (0.5 mL) previously heated at 140 °C. The resulting solution was stirred for 10 h at this temperature and washed with saturated aqNaCl, extracted with ethyl acetate (3 × 15 mL). The combined organic phase was dried with anhydrous Na_2_SO_4_, filtrated and concentrated under vacuum to yield the crude product. The crude product was purified by thin layer chromatography on silica gel with petroleum ether as eluent.

### 3.3. Analytical Data of Representative Products

*2,3,6-Triphenylpyridine*: White crystals (m.p. = 110–111 °C, lit [24] 110.5–112 °C, lit [25] 111–112 °C). ^1^H-NMR (400 MHz, CDCl_3_) δ 8.20 (d, 2H), 7.98–7.75 (m, 2H), 7.50 (dq, 5H), 7.30 (ddd, 8H).^13^C-NMR (101 MHz, CDCl_3_) δ 156.64, 155.68, 140.43, 140.01, 139.43, 139.10, 134.43, 130.23, 129.59, 129.01, 128.75, 128.37, 127.84, 127.18, 127.02, 118.59. lit [25]: ^1^H-NMR (400MHz, CDCl_3_) δ 8.16–8.14 (m, 2H), 7.78–7.77 (m, 2H), 7.51–7.42 (m, 5H), 7.30–7.21 (m, 9H); ^13^C-NMR (100 MHz, CDCl_3_) δ 156.6, 155.6, 140.4, 140.0, 139.4, 139.1, 134.4, 130.2, 129.5, 129.0, 128.7, 128.3, 127.8, 127.1, 127.0, 118.5. HRMS (EI) calcd. for C_23_H_17_N: 307.1361, found: 307.2. 

*2-(4-Fluorophenyl)-3,6-diphenylpyridine*: White solid (m.p. = 115–117 °C, lit [22] 115–116 °C). ^1^H-NMR (400 MHz, CDCl_3_) δ 8.19 (d, 2H), 7.82 (d, 2H), 7.52 (dd, 5H), 7.32 (d, 3H), 7.27 (d, 2H), 7.01 (d, 2H). ^13^C-NMR (101 MHz, CDCl_3_) δ 162.53 (*J_C−F_* = 245.6 Hz), 155.73, 155.53, 139.81, 139.56, 138.95, 134.31 (*J_C−F_* = 4.3 Hz), 132.06, 131.97 (*J_C−F_* = 8.2 Hz), 129.55, 129.13, 128.82, 128.53, 127.34, 126.99, 118.70, 114.74(*J_C−F_* = 21.5 Hz). lit [22]: ^1^H-NMR (400 MHz, CDCl_3_): δ 8.13 (d, 2H), 7.77 (s, 2H), 7.51–7.42 (m, 5H), 7.31–7.29 (m, 3H), 7.22–7.20 (m, 2H), 6.94 (t, 2H); ^13^C-NMR (100 MHz, CDCl_3_): 162.5 (*J_C−F_* = 245.6 Hz), 155.7, 155.5, 139.8, 139.5, 138.9, 136.4 (*J_C−F_* = 4.3 Hz), 134.2, 131.9 (*J_C−F_* = 8.2 Hz), 129.5, 129.0, 128.7, 128.4, 127.2, 126.9, 118.6, 114.7 (*J_C−F_*= 21.5Hz). HRMS (EI) calcd. for C_23_H_16_FN: 325.1267, found: 325.2.

## 4. Conclusions

In summary, an efficient protocol for arylated pyridines from conjugated acetylenes and substituted benzylamines catalyzed by base was developed, which gives a much more convenient approach to obtain arylated pyridines with good to excellent yields. Compared to the approachreported by Chalk [21], the advantages of this protocol are inthe absence ofbyproduct detected by GC-MS even if the reaction was carried out in the air. Efforts to understand this reaction mechanism are in progress in our laboratory.

## Figures and Tables

**Table 1:**
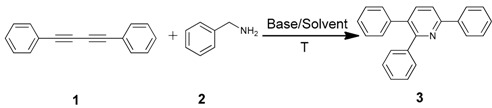
Optimization of the reaction conditions ^a^.

Entry	Ratio of 1:2	Temperature	Solvent	Catalyst	Yield(%) ^b^
1	1:10	140 °C	DMSO	K_2_CO_3_	96
2	1:8	140 °C	DMSO	K_2_CO_3_	96
3	1:6	140 °C	DMSO	K_2_CO_3_	85
4	1:5	140 °C	DMSO	K_2_CO_3_	80
5	1:4	140 °C	DMSO	K_2_CO_3_	70
6	1:8	120 °C	DMSO	K_2_CO_3_	70
7	1:8	80 °C	DMSO	K_2_CO_3_	30
8	1:8	140 °C	DMF	K_2_CO_3_	99
9	1:8	140 °C	DMAc	K_2_CO_3_	94
10	1:8	140 °C	PEG400	K_2_CO_3_	50
11	1:8	140 °C	DMF	_	38
12	1:8	140 °C	DMF	Cs_2_CO_3_	65
13	1:8	140 °C	DMF	Na_2_CO_3_	81
14	1:8	140 °C	DMF	NaOH	86
15	1:8	140 °C	DMF	KOH	88
16	1:8	140 °C	DMF	NaF	65
17	1:8	140 °C	DMF	NaHCO_3_	87
18	1:8	140 °C	DMF	NaH_2_PO_4_	53
19	1:8	140 °C	DMF	KH_2_PO_4_	61
20	1:8	140 °C	DMF	CH_3_COONa	63

^a^ The reactions were conducted with 1,4-diphenylbutadiyne and benzylamine, and base (0.5 mmol), solvent (0.5 mL), 10 h; ^b^ Isolated yield.

**Table 2:**
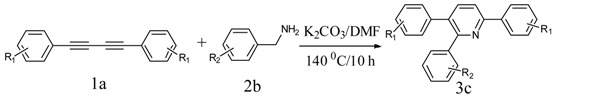
Synthesis of arylated pyridines from conjugated acetylenes and substituted benzylamines under optimized conditions. ^a^

Entry	Acetylene	Benzylamine	Product	Yield(%) ^b^
1	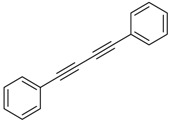	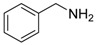	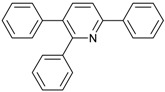 **3cab**	99
2	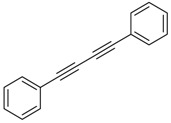	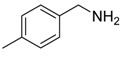	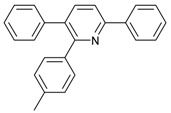 **3cac**	99
3	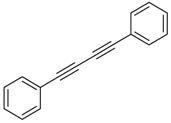	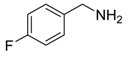	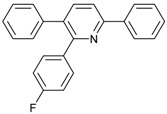 **3cad**	73
4	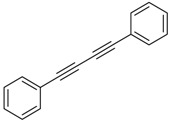	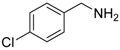	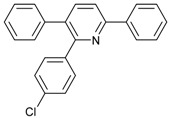 **3cae**	62
5	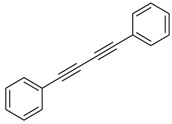	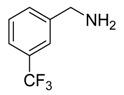	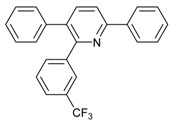 **3caf**	50
6	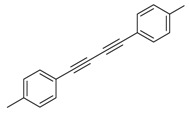	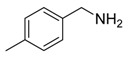	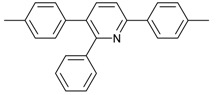 **3cbb**	90
7	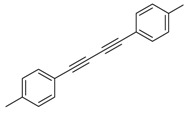	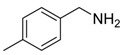	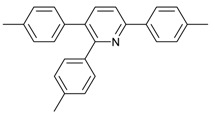 **3cbc**	99
8	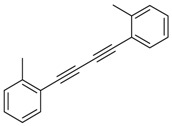	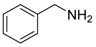	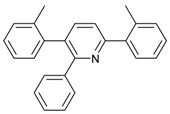 **3ccb**	77
9	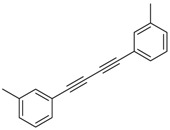	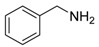	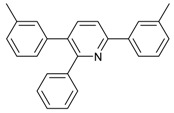 **3cdb**	78
10	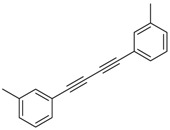	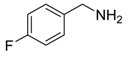	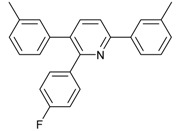 **3ccd**	63
11	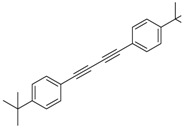	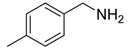	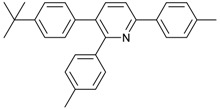 **3cec**	65
12	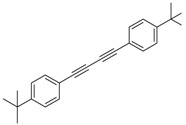	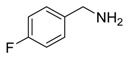	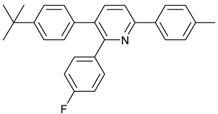 **3ced**	45
13	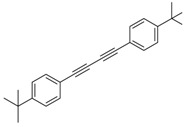	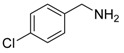	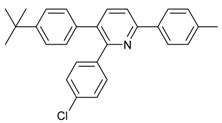 **3cee**	48
14	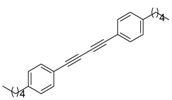	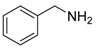	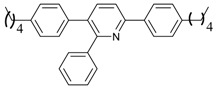 **3cfb**	60

^a^ Reaction conditions: conjugated acetylene (**1a**) (0.25 mmol), substituted benzylamine (**2b**) (2.0 mmol), K_2_CO_3_ (0.5 mmol), DMF (0.5 mL), 140 °C, 10 h; ^b^ Isolated yield.

## Supplementary Materials

Supplementary materials are available online.
