# Supplementary material for: An Efficient Synthesis of Arylated Pyridines from Conjugated Acetylenes and Substituted Benzylamines Catalyzed by Base
Source: Molecules. 2017 Jul 31;22(8):1277. doi: 10.3390/molecules22081277 (PMC6152059; doi:10.3390/molecules22081277)

Supplementary Materials

An efficient synthesis of arylated pyridines from conjugated acetylenes and substituted benzylamines catalyzed by base

Mengping Guo, Bo Chen, Qiming Zhu, Hua Jin, Qiuling Peng, Yanping Kang

Characterization

1. ^{1}H NMR and ^{13}C NMR of 2,3,6-triphenylpyridine

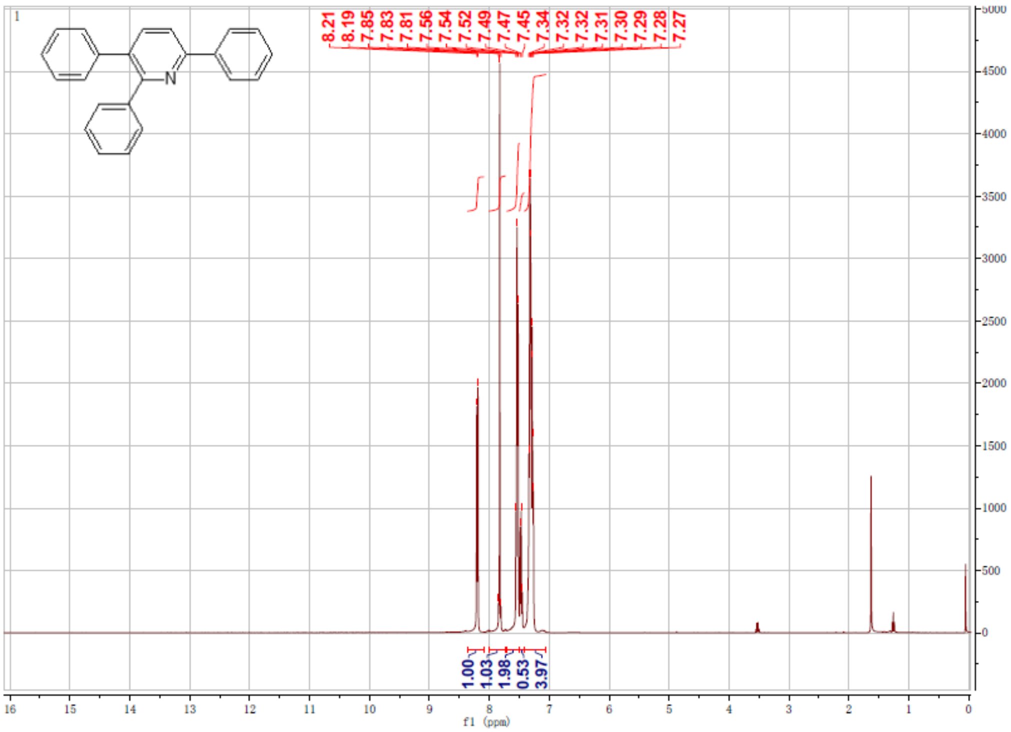

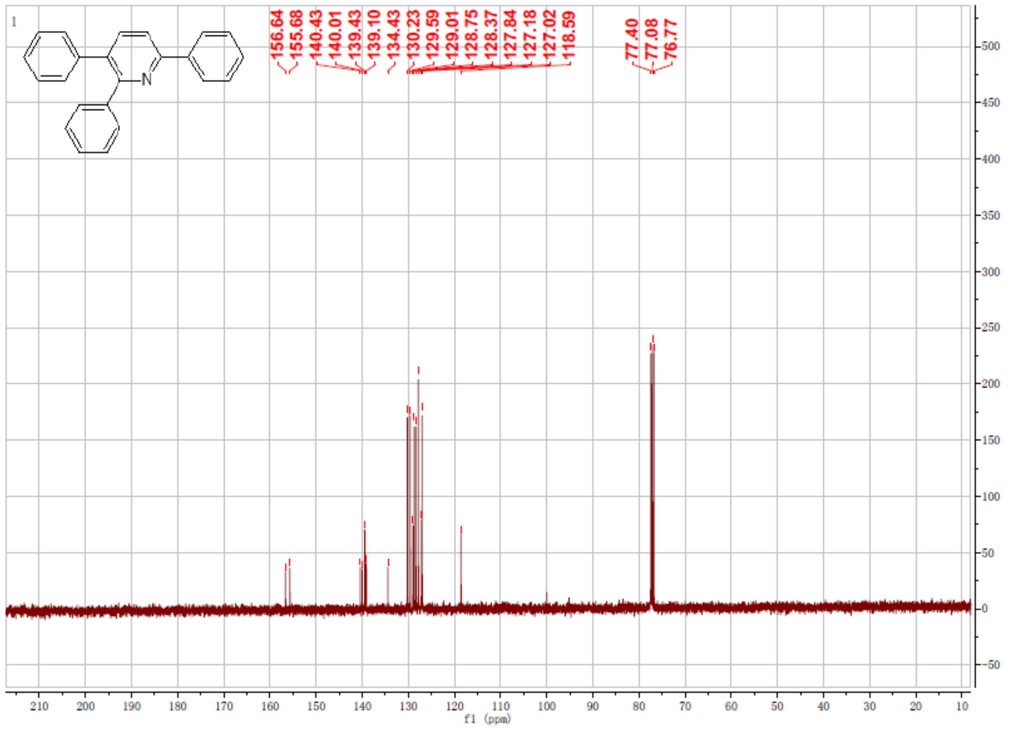

^{1}H NMR and ^{13}C NMR of 3,6-diphenyl-2-(p-toly)pyridine

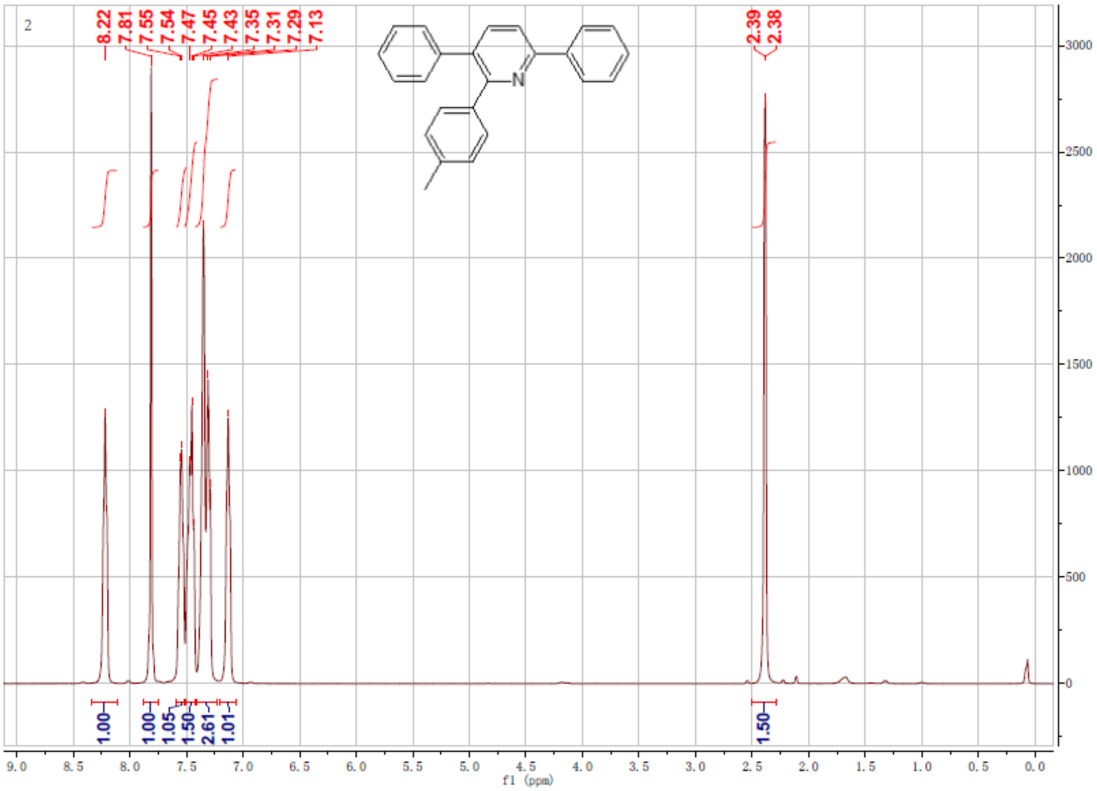

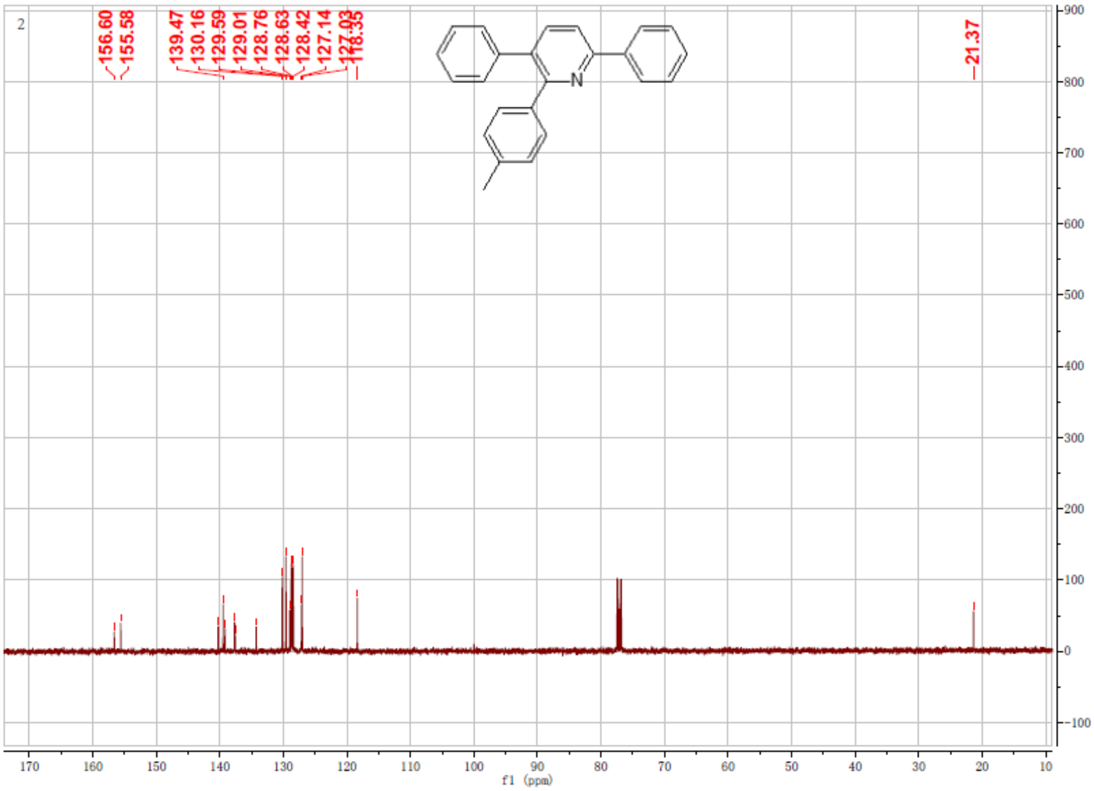

^{1}H NMR and ^{13}C NMR of 2-(4-fluorophenyl)-3,6-diphenylpyridine

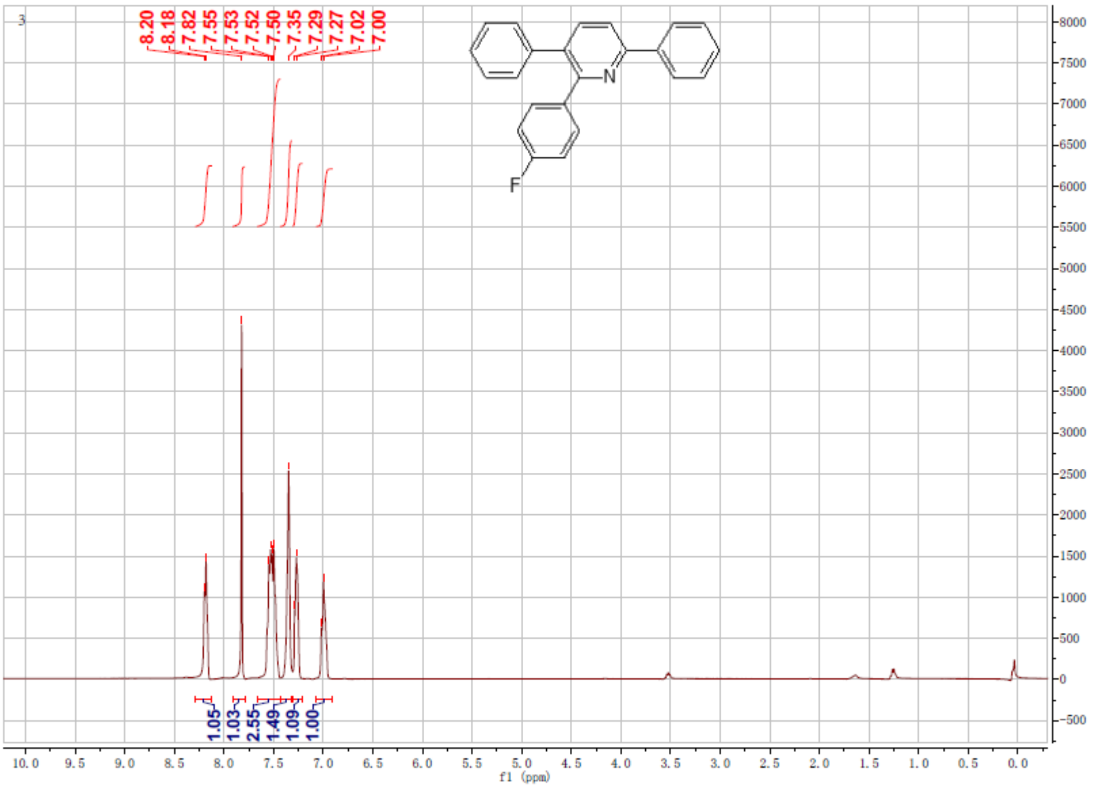

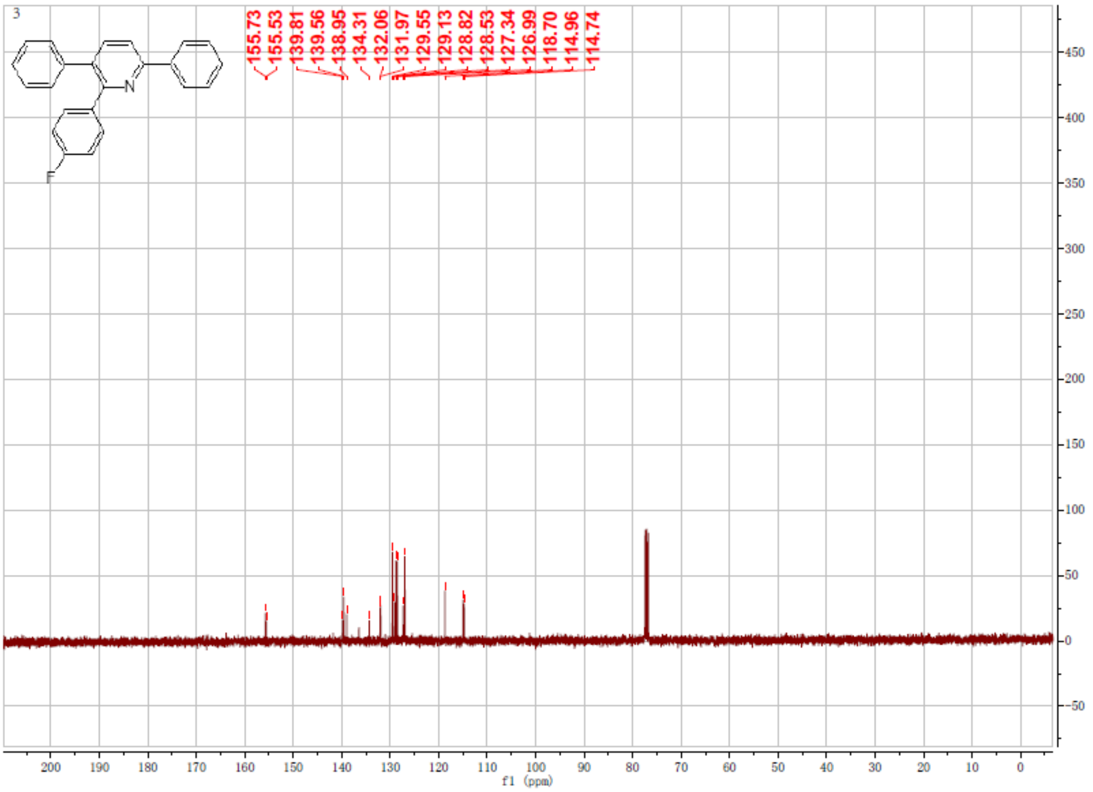

^{1}H NMR and ^{13}C NMR of 2-(4-chlorophenyl)-3,6-diphenylpyridine

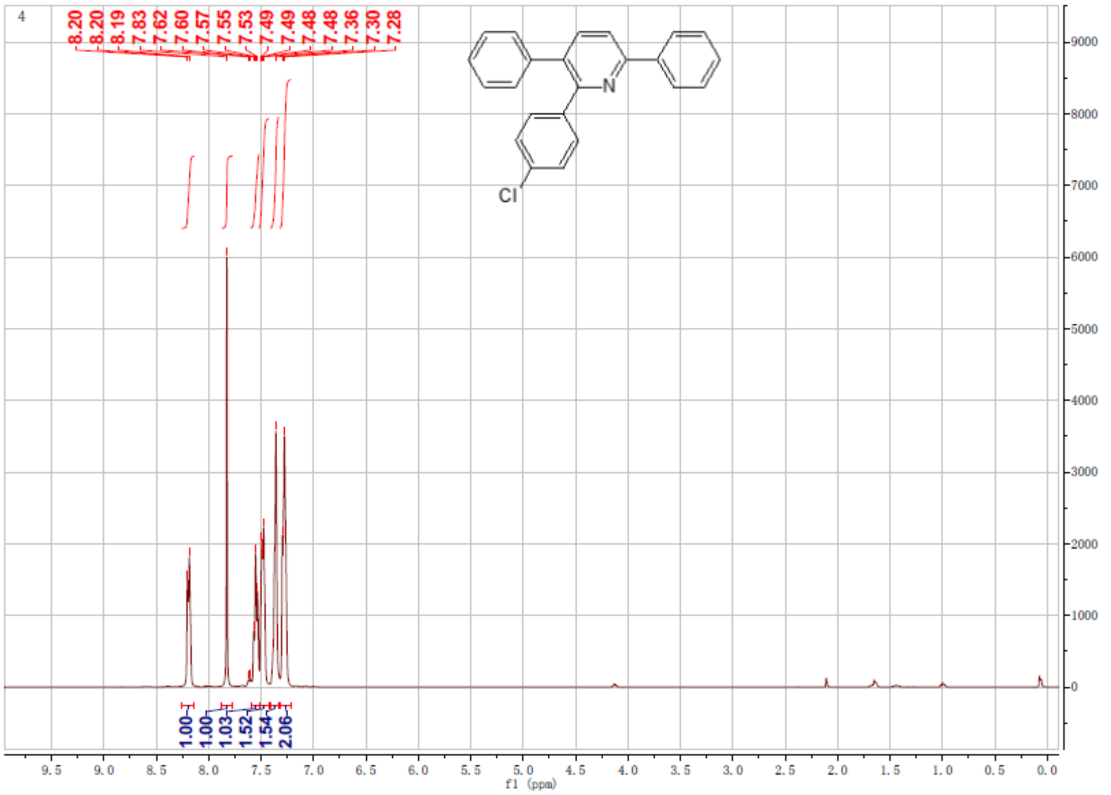

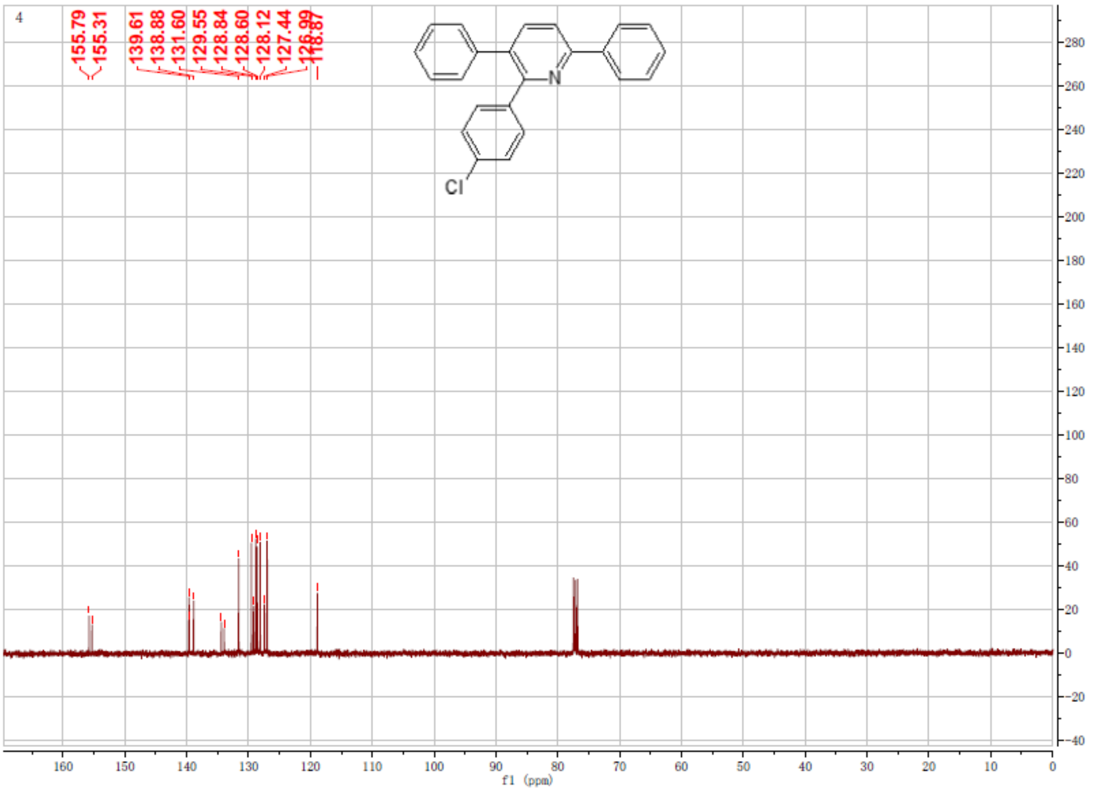

^{1}H NMR and ^{13}C NMR of 3,6-diphenyl-2-(3-(trifluoromethyl)phenyl)pyridine

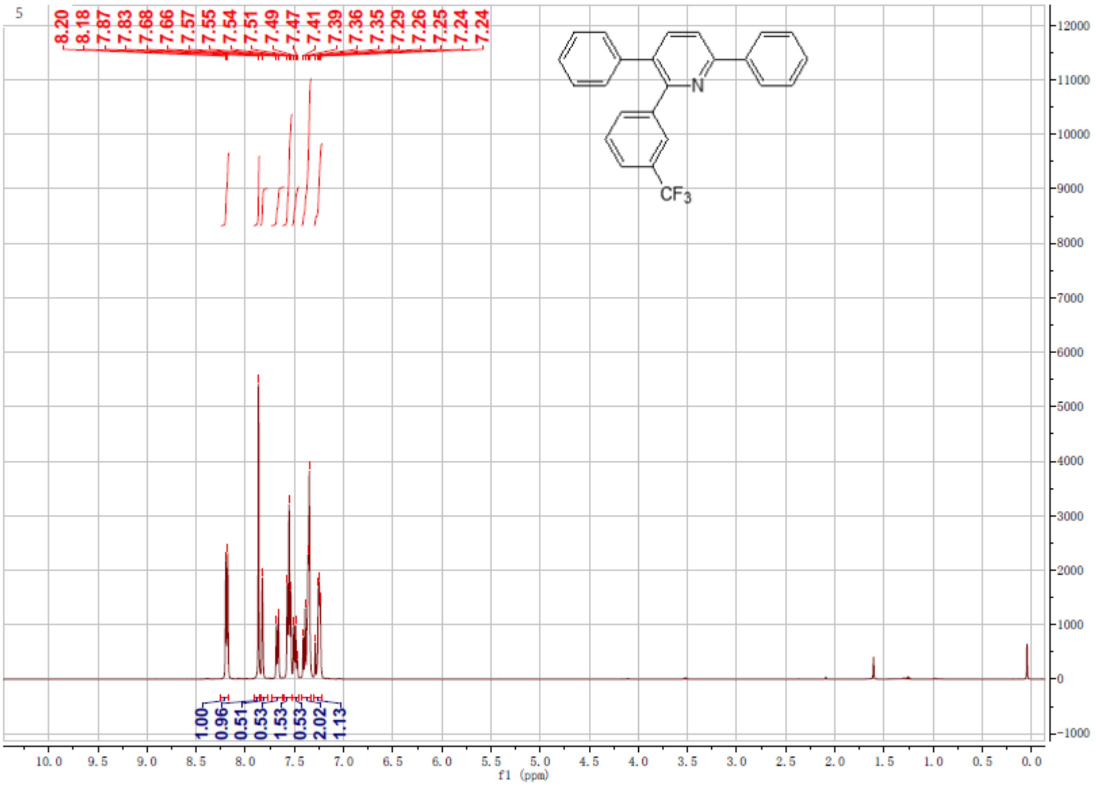

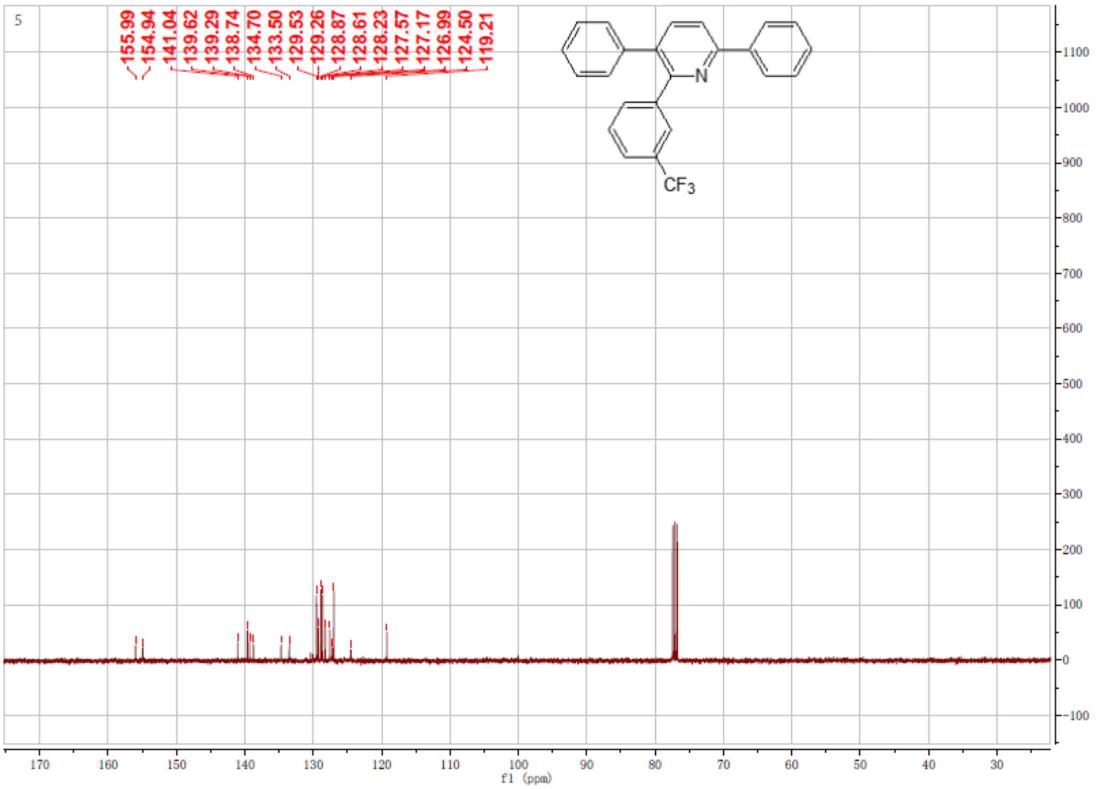

^{1}H NMR and ^{13}C NMR of 2-phenyl-3,6-di-p-tolylpyridine

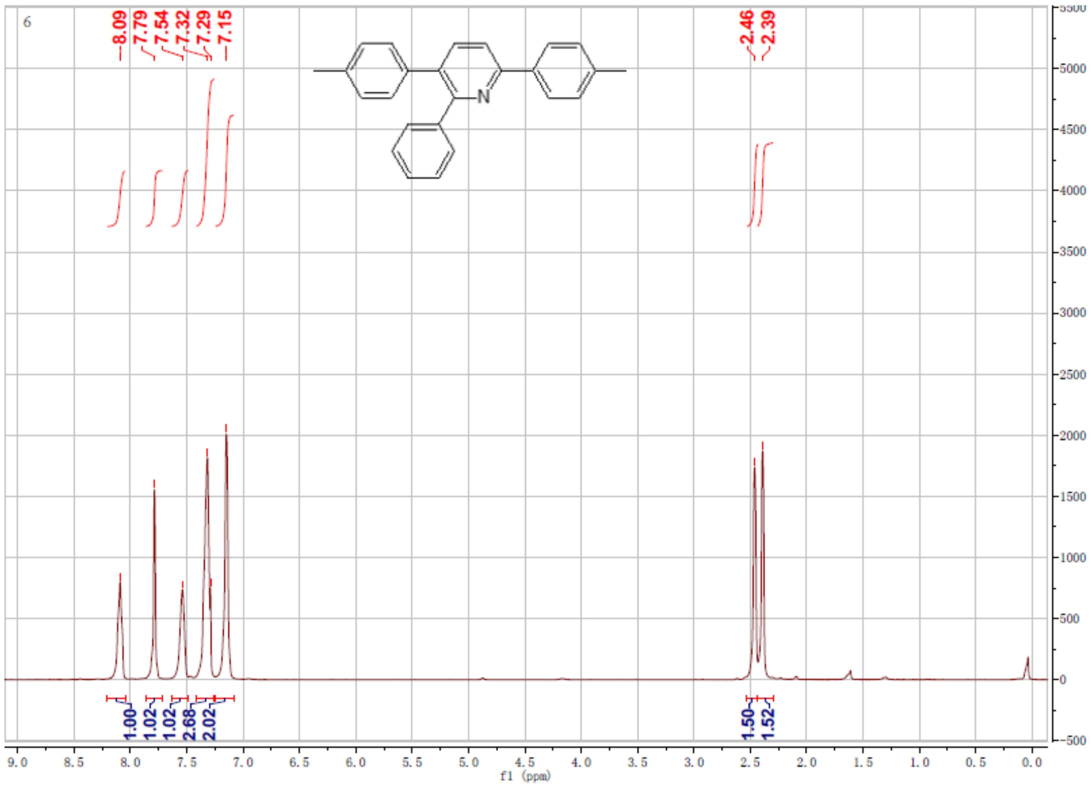

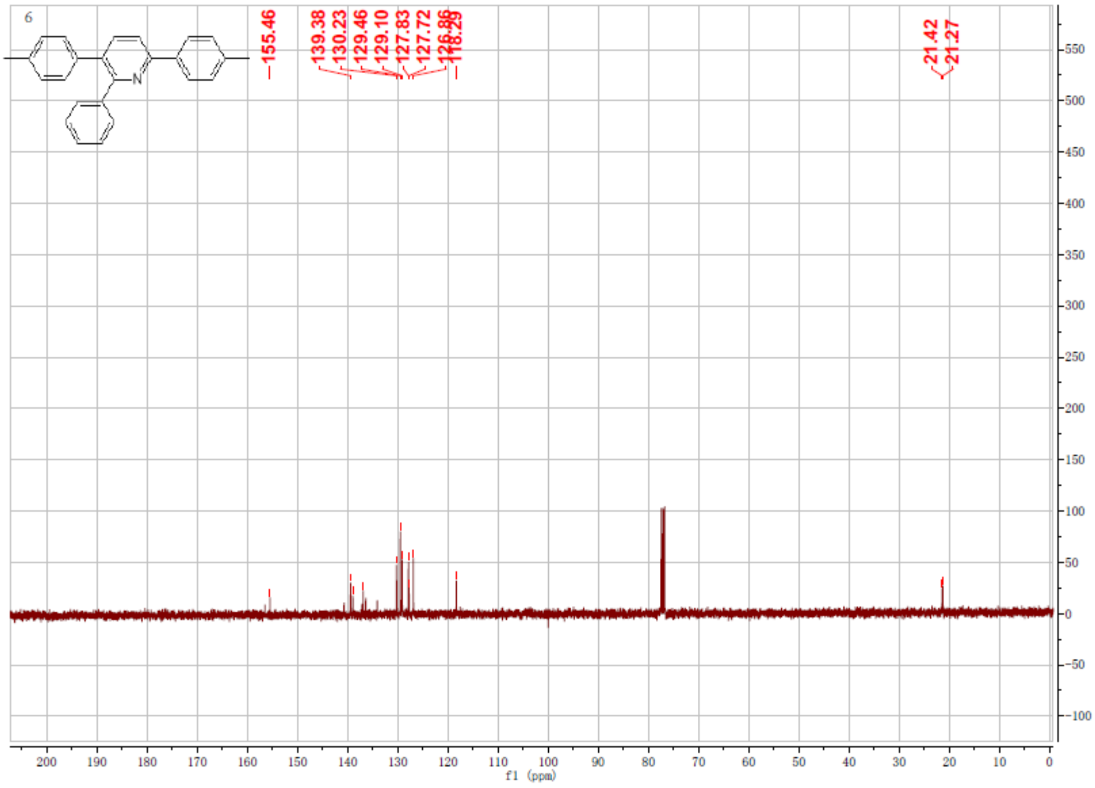

^{1}H NMR and ^{13}C NMR of 2,3,6-tri-p-tolylpyridine

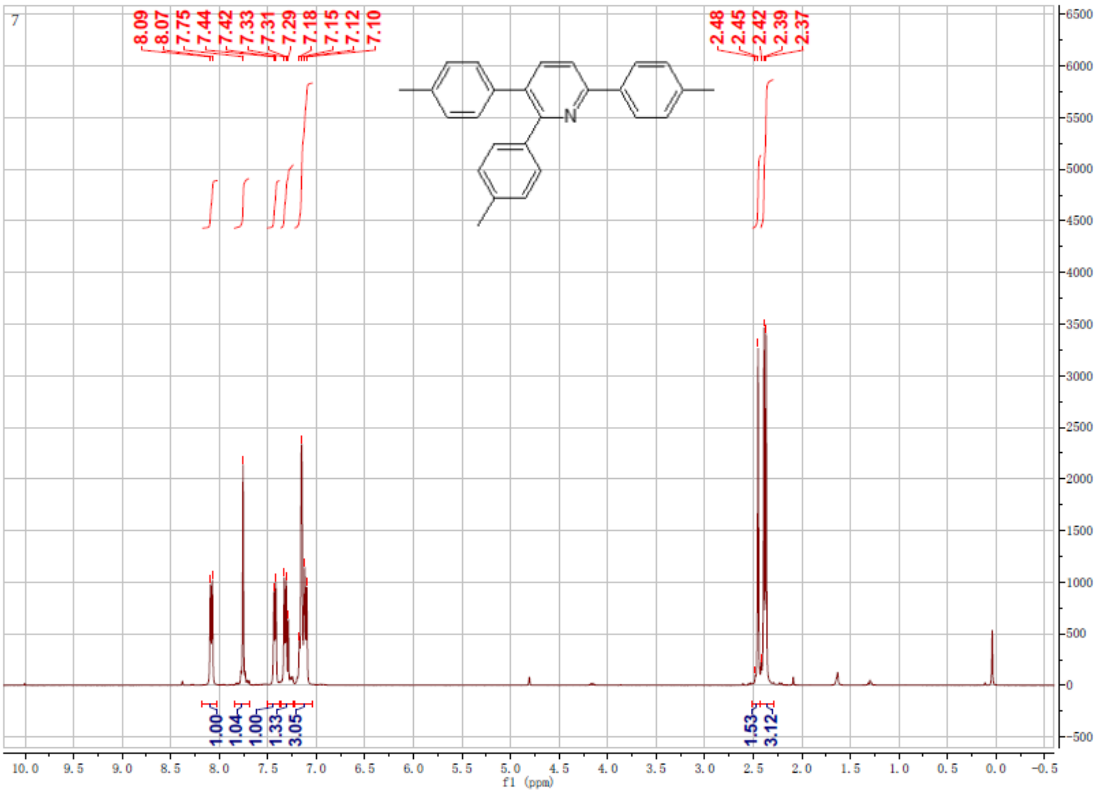

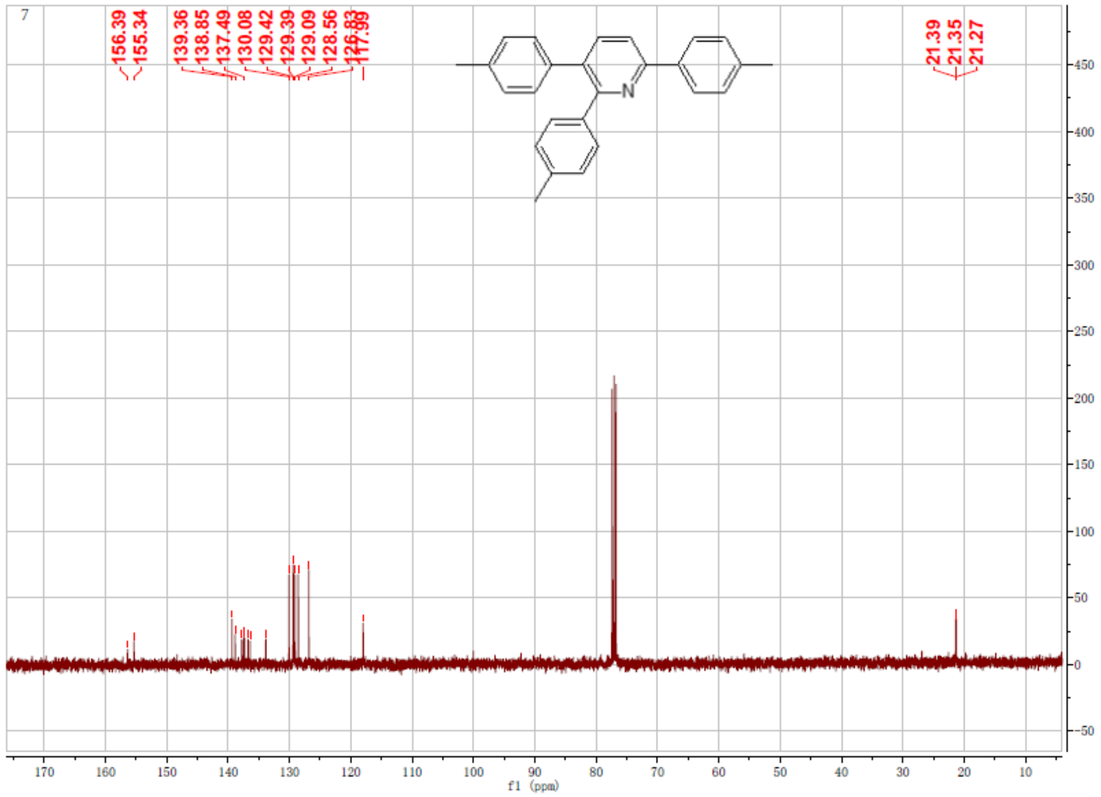

^{1}H NMR and ^{13}C NMR of 2-phenyl-3,6-di-m-tolylpyridine

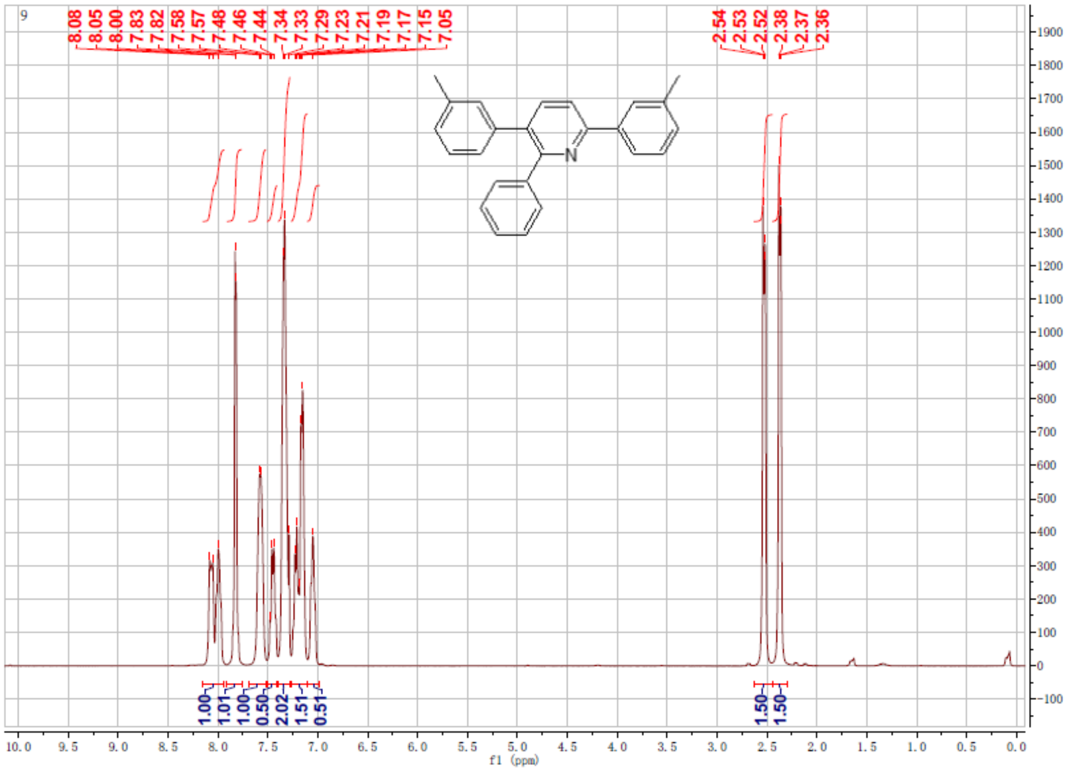

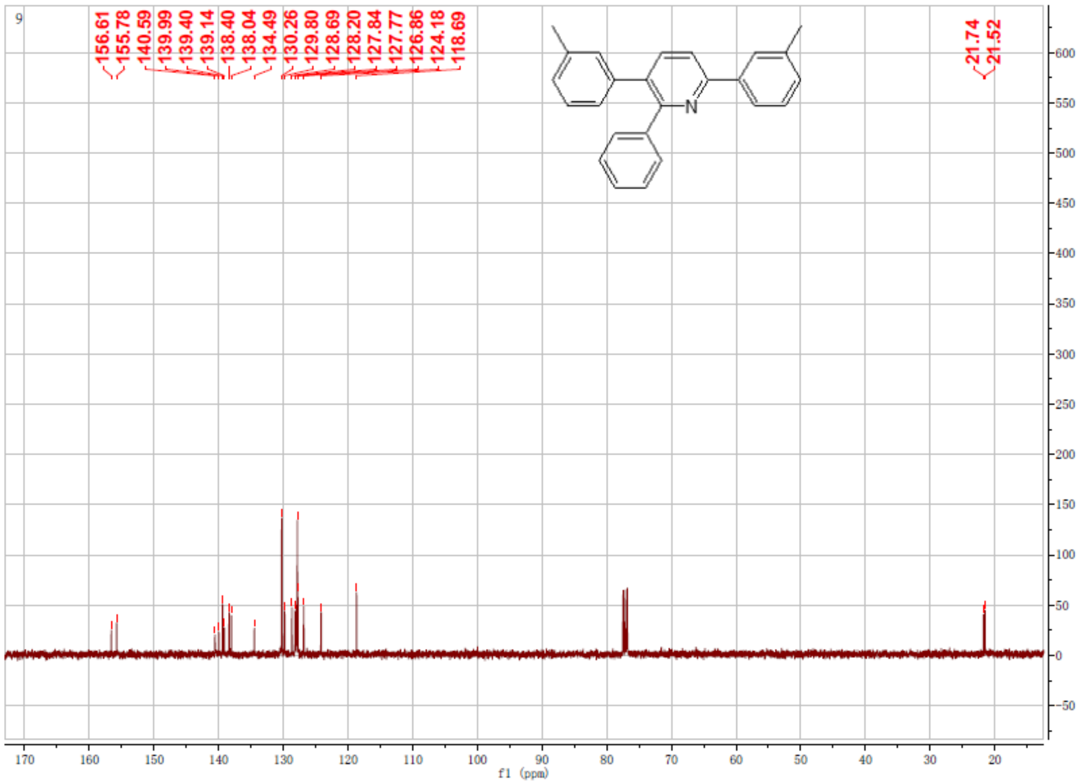

¹H NMR and ¹³C NMR of 3,6-bis(4-butylphenyl)-2-phenylpyridine

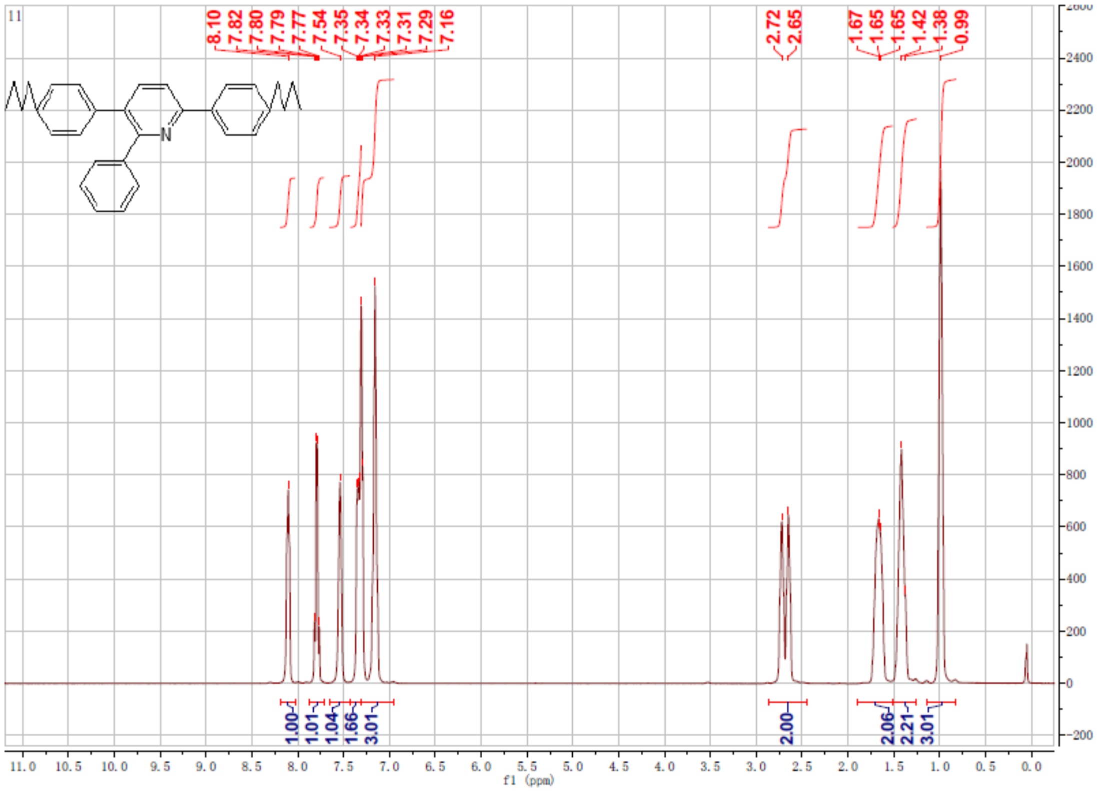

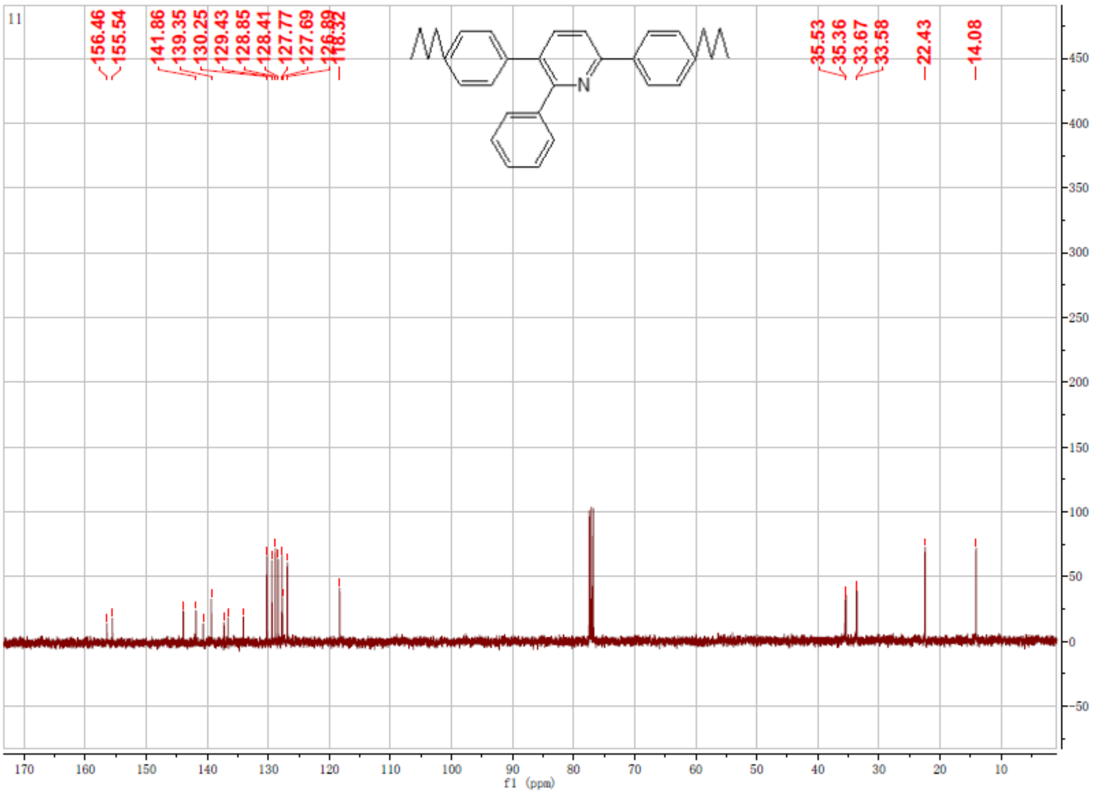

^{1}H NMR and ^{13}C NMR of 3,6-bis(4-(tert-butyl)phenyl)-2-(p-tolyl)pyridine

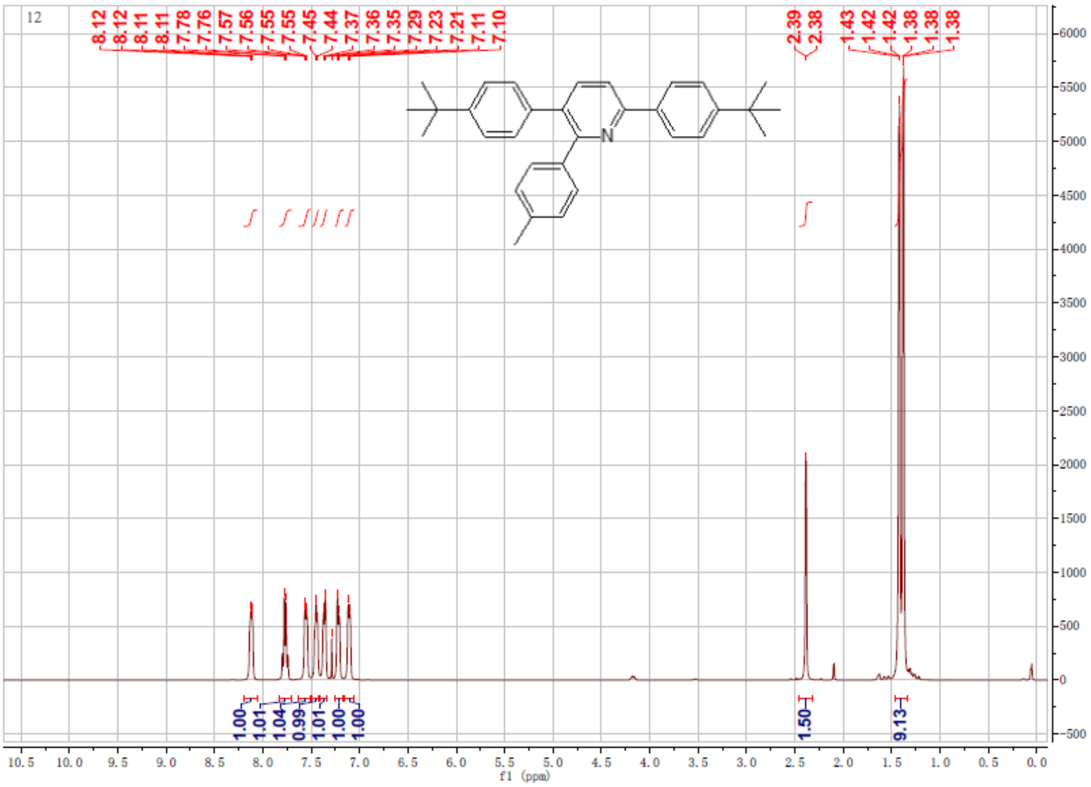

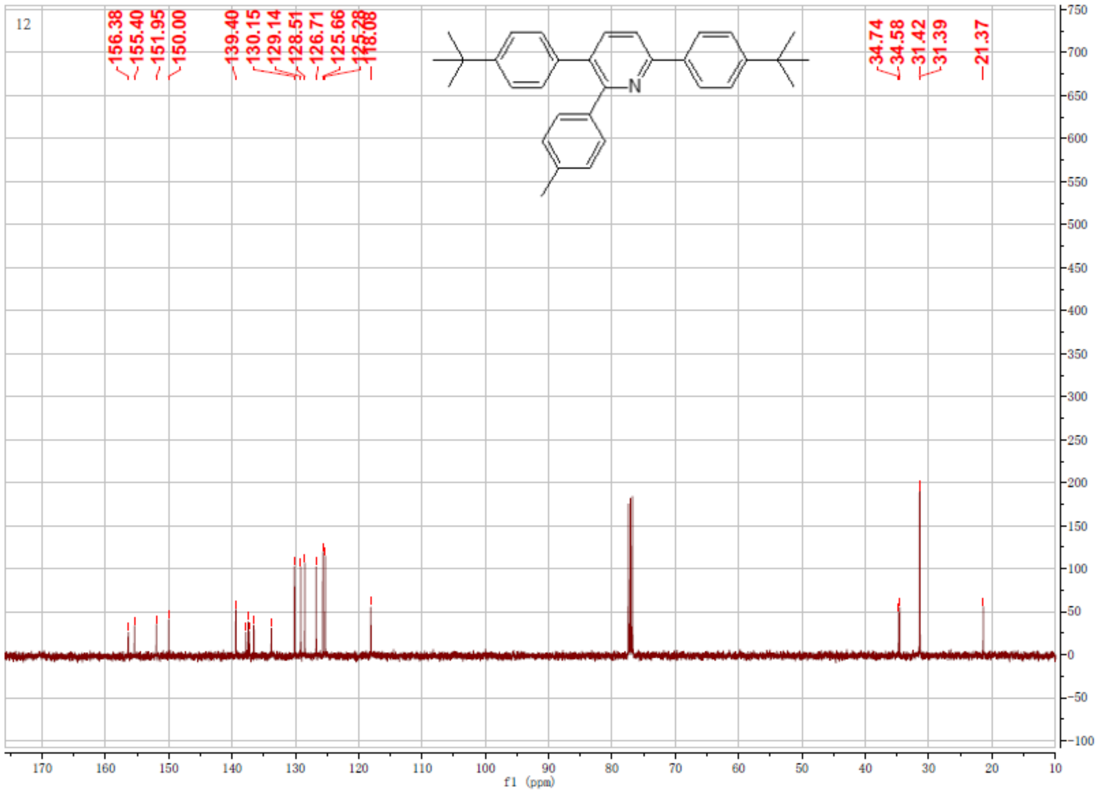

^{1}H NMR and ^{13}C NMR of 3,6-bis(4-(tert-butyl)phenyl)-2-(4-fluorophenyl)pyridine

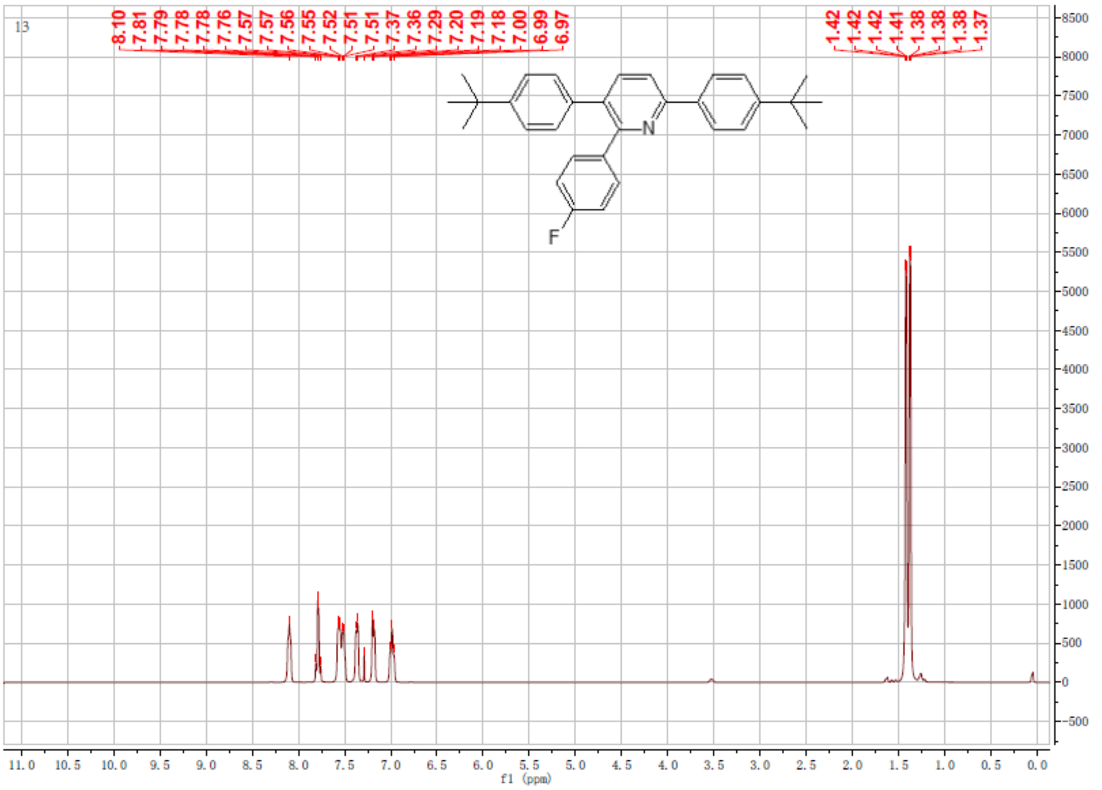

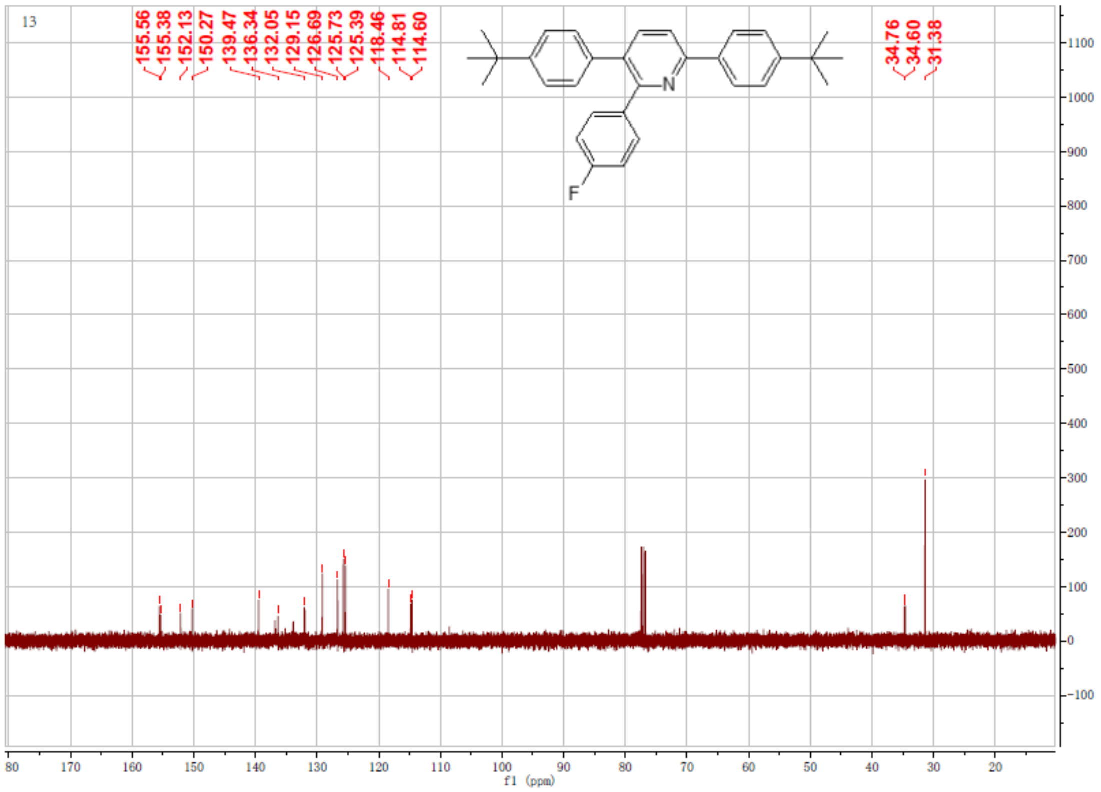

2. HRMS of the arylated pyridines

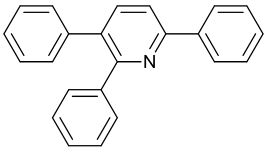

calcd for $C_{23}H_{17}N$: 307.1361, found: 307.2.

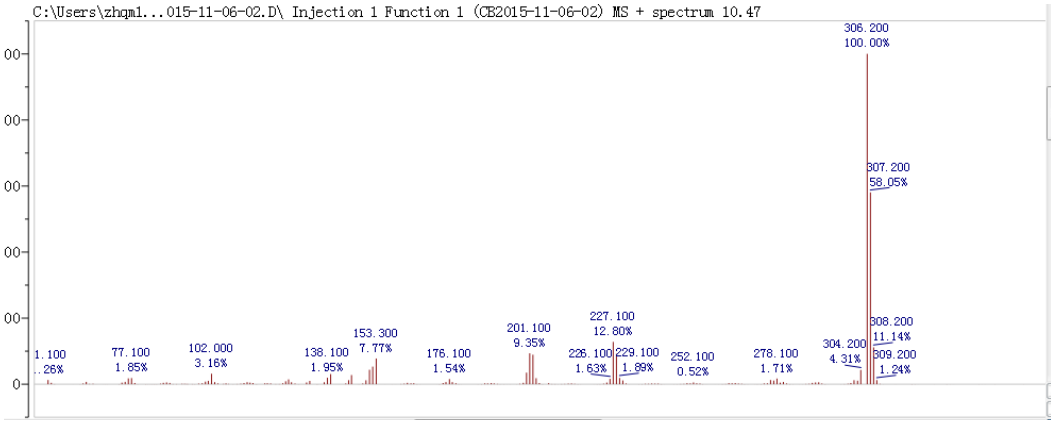

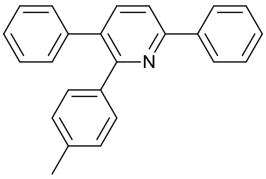

calcd for $C_{24}H_{19}N$: 321.1517, found: 321.2.

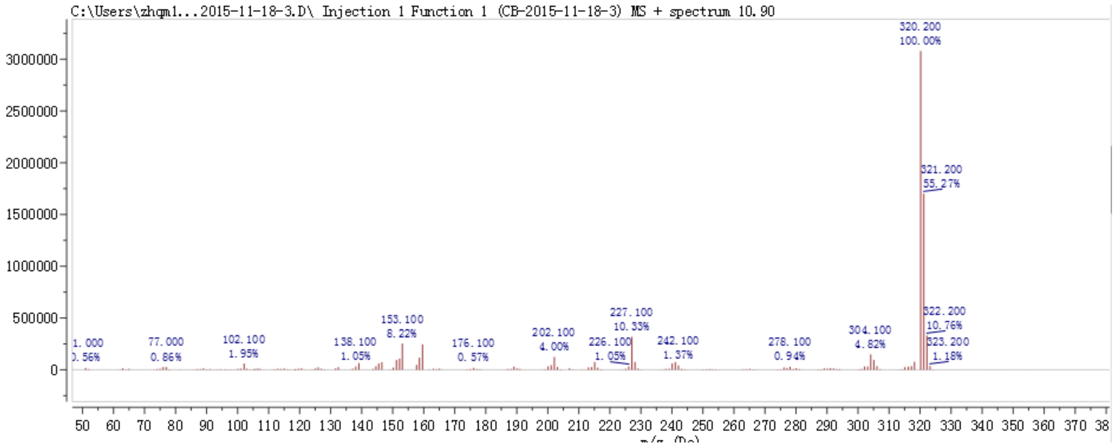

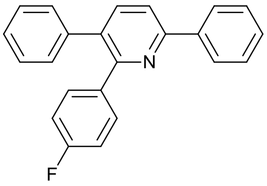

calcd for $C_{23}H_{16}N$: 325.1267, found: 325.2.

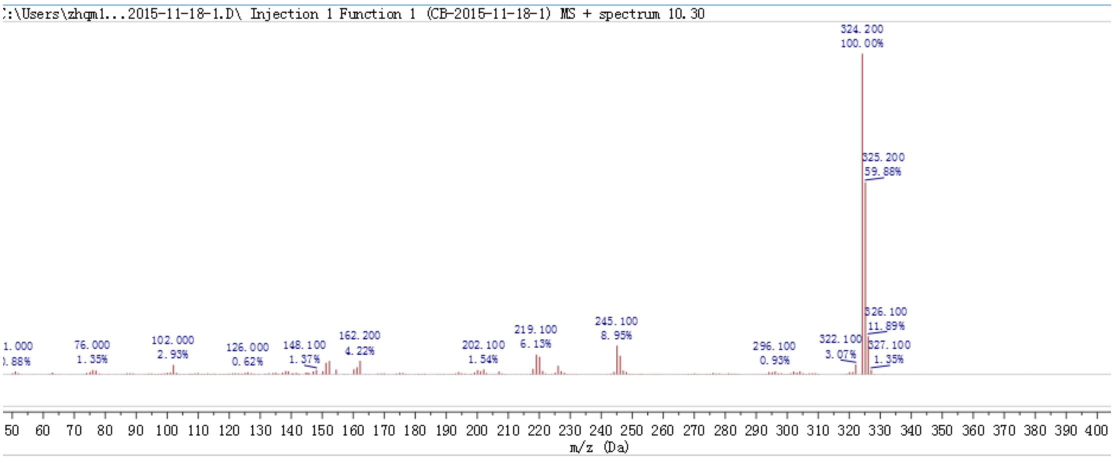

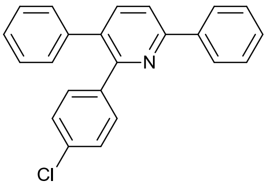

calcd for $C_{23}H_{16}ClN$: 341.0971, found: 341.1.

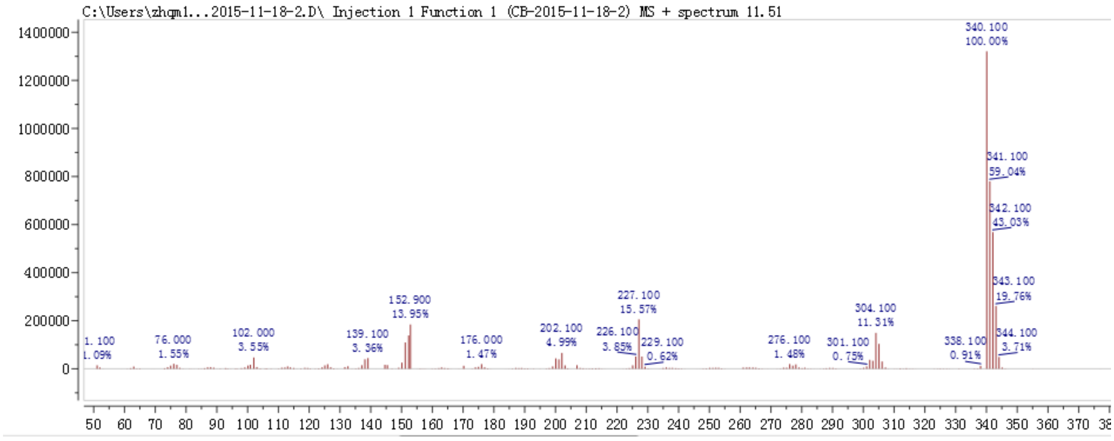

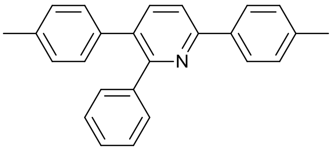

calcd for $C_{25}H_{21}N$: 335.1674, found: 335.2.

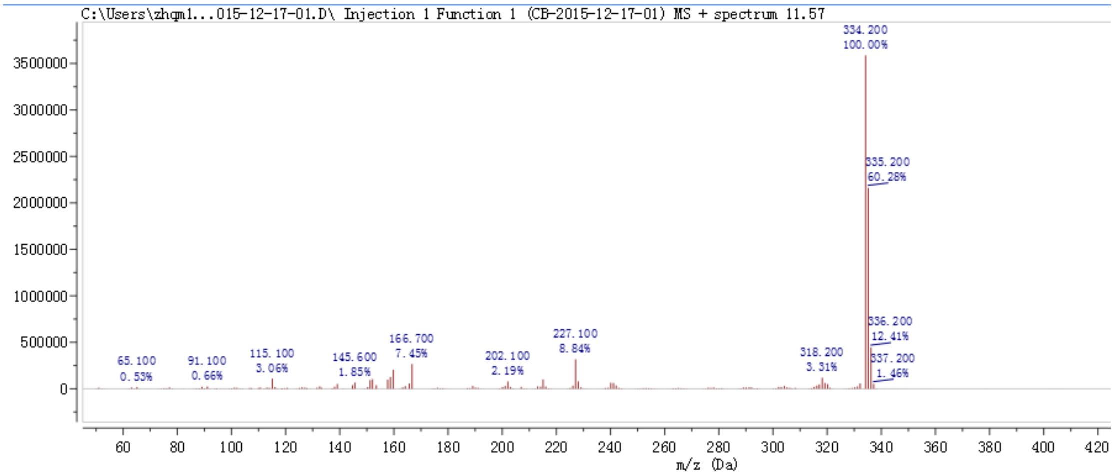

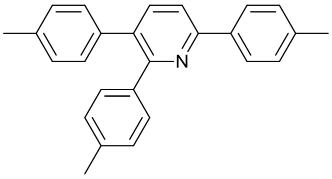

calcd for $C_{26}H_{23}N$: 349.1830, found: 349.2.

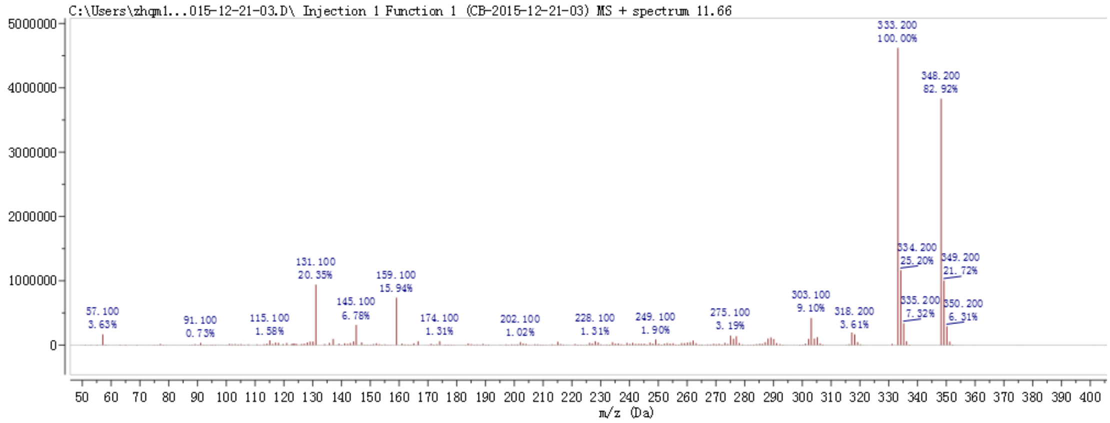

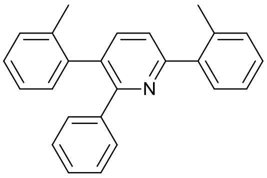

calcd for $C_{26}H_{23}N$: 335.1674, found: 335.2.

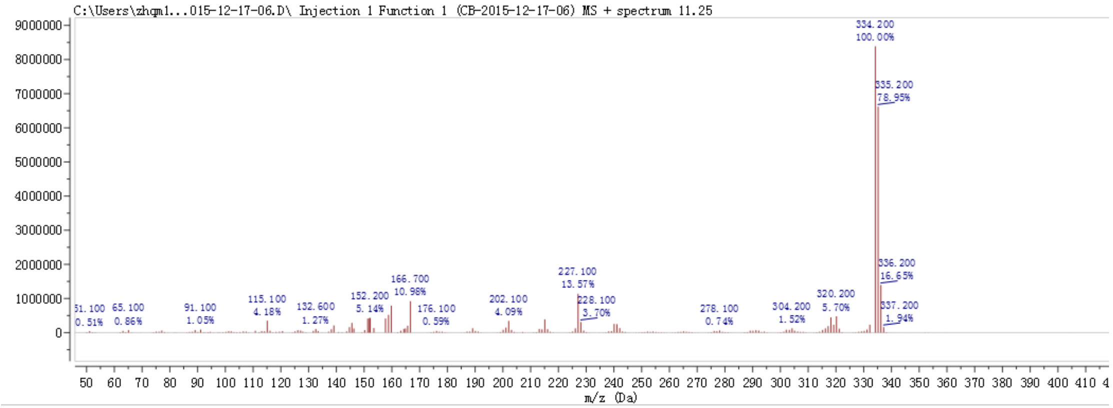

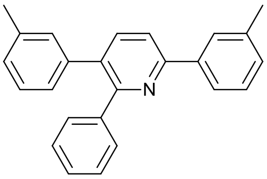

calcd for $C_{26}H_{23}N$: 335.1674, found: 335.2.

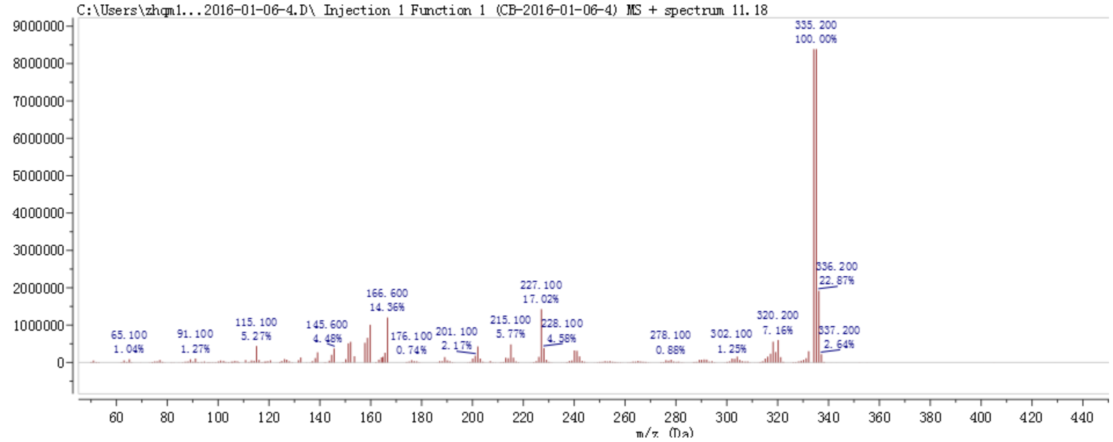

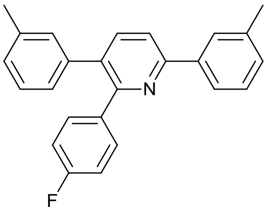

calcd for $C_{25}H_{20}FN$: 353.1580, found: 353.2.

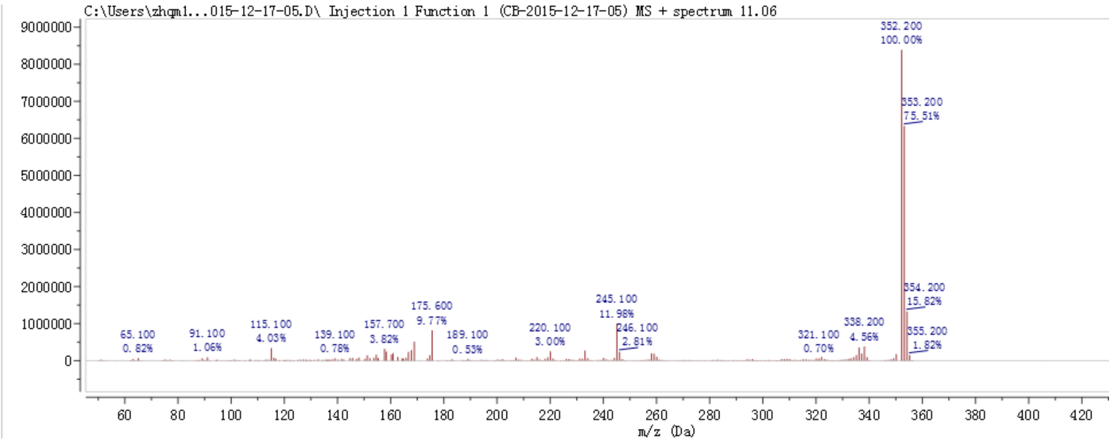

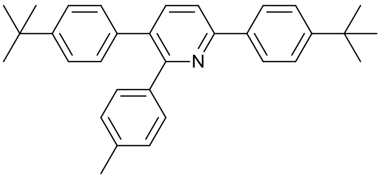

calcd for $C_{32}H_{35}N$: 433.2770, found: 433.3

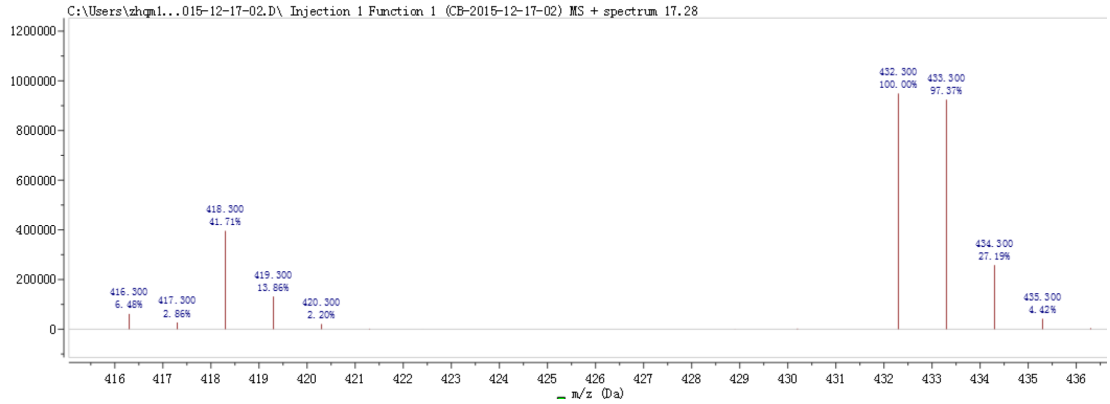

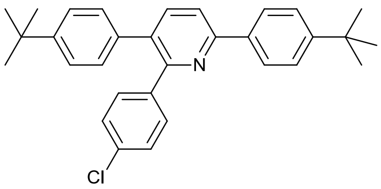

calcd for $C_{31}H_{32}ClN$: 453.2223, found: 453.3

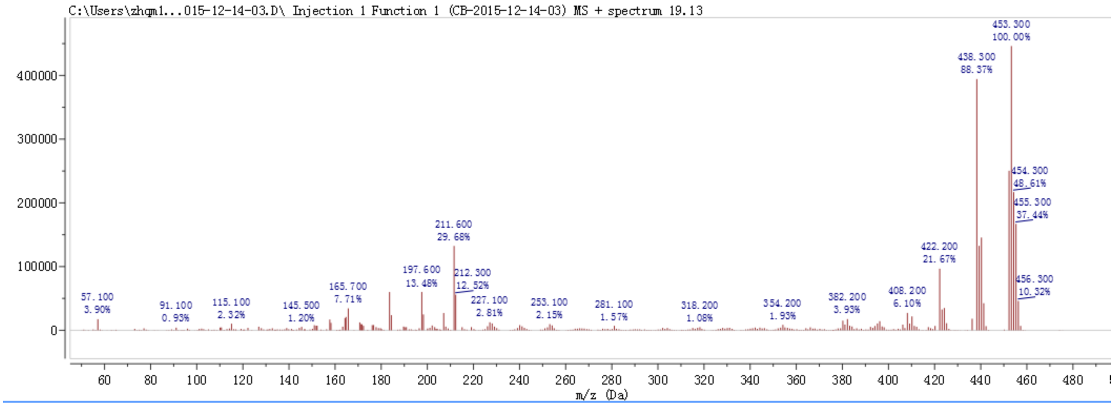

Supplement: Supplementary file 1 [file molecules-22-01277-s001.pdf]
